# Optical properties of plasmonic nanopore arrays prepared by electron beam and colloidal lithography†

**DOI:** 10.1039/c9na00585d

**Published:** 2019-10-07

**Authors:** Bita Malekian, Kunli Xiong, Evan S. H. Kang, John Andersson, Gustav Emilsson, Marcus Rommel, Takumi Sannomiya, Magnus P. Jonsson, Andreas Dahlin

**Affiliations:** Department of Chemistry and Chemical Engineering, Chalmers University of Technology 41296 Gothenburg Sweden adahlin@chalmers.se; Laboratory of Organic Electronics, Linköping University 60174 Norrköping Sweden; Department of Microtechnology and Nanoscience, Chalmers University of Technology 41296 Gothenburg Sweden; Department of Materials Science and Engineering 4259 Nagatsuta Midoriku Yokohama 226-8503 Japan

## Abstract

Solid state nanopores are central structures for many applications. To date, much effort has been spent on controlled fabrication of single nanopores, while relatively little work has focused on large scale fabrication of arrays of nanopores. In this work we show wafer-scale fabrication of plasmonic nanopores in 50 nm thick silicon nitride membranes with one or two 30 nm gold films, using electron beam lithography with a negative resist or a new version of colloidal lithography. Both approaches offer good control of pore diameter (even below 100 nm) and with high yield (>90%) of intact membranes. Colloidal lithography has the advantage of parallel patterning without expensive equipment. Despite its serial nature, electron beam lithography provides high throughput and can make arbitrary array patterns. Importantly, both methods prevent metal from ending up on the membrane pore sidewalls. The new fabrication methods make it possible to compare the optical properties of structurally identical plasmonic nanopore arrays with either long-range order (e-beam) or short-range order (colloidal). The resonance features in the extinction spectrum are very similar for both structures when the pitch is the same as the characteristic spacing in the self-assembled colloidal pattern. Long-range ordering slightly enhances the magnitude of the extinction maximum and blueshift the transmission maximum by tens of nm. Upon reducing the diameter in long-range ordered arrays, the resonance is reduced in magnitude and the transmission maximum is further blue shifted, just like for short-range ordered arrays. These effects are well explained by interpreting the spectra as Fano interference between the grating-type excitation of propagating surface plasmons and the broad transmission *via* individual pores in the metal film. Furthermore, we find that only the short-range ordered arrays scatter light, which we attribute to the highly limited effective period in the short-range ordered system and the corresponding lack of coherent suppression of scattering by interference effects.

## Abstract 2

We present new plasmonic nanopore arrays and their optical properties, in particular the influence from short-range *vs.* long-range ordering.

## Graphical abstract

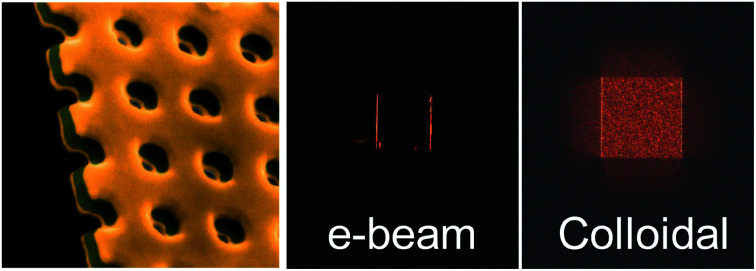

## Introduction

Nanoscale apertures in thin membranes are of great interest in nanotechnology. Much effort has been spent on advanced fabrication of individual nanopores for single molecule experiments.^1^ In most cases, thin silicon nitride membranes supported by a silicon wafer are utilized because of their ease of preparation and relatively high stability. Recently, there has also been a strong interest in nanopores with additional nanostructures in metals on the membranes.^2^ Although analysis of single (or few) nanopores is necessary for detection of individual molecules by ion current measurements, arrays of many pores in thin membranes enable other applications. For instance, a single pore cannot be used as an efficient filter, while arrays of pores in thin membranes enable biomolecular filtration with high diffusive flux.^3–5^ Furthermore, when arrays of apertures are present in thin metal films, excitation of surface plasmons is possible,^6^ which enables refractometric sensing in combination with efficient delivery by the flow-through configuration.^7^ Pores in continuous metal films are also useful for implementing electrochemistry,^8^ dielectrophoresis^9^ or temperature control.^10^ In addition, material-specific surface chemistry (*e.g.* thiols on gold) can be used to selectively bind molecules to certain regions of the nanostructure.^11^

However, several challenges have not been addressed when it comes to controlled and reliable fabrication of nanopore arrays in continuous metal films. Many advanced methods used to prepare single nanopores are often not feasible to upscale for making arrays. One option is to use photolithography, but this limits the aperture diameter to at least hundreds of nm.^12^ Electron beam lithography (EBL) offers high resolution, but has mainly been used to prepare nanopores using positive resists.^13^ Importantly, if a metallic coating is to be included with such fabrication approaches, the metal tends to be deposited onto existing pores in a (dielectric) membrane. This makes it difficult to avoid metal residues on the preexisting pore walls since the directionality is never perfect.^14,15^ This, in turn, limits control of the fine structure and complicates selective chemical functionalization of the pore interior. One option to circumvent this problem is colloidal lithography (CL), which has been used to prepare plasmonic nanopores (or nanoholes) in short-range as well as hexagonal long-range order.^10,16–22^ Unfortunately, arbitrary patterns are not possible with CL and the process puts high demands on membrane stability in order to keep them intact during lift-off.^17^ Fabrication aspects aside, there is a strong interest in understanding how the ordering of the apertures influences the optical response.^18,19,23,24^ In fact, several aspects of the optical properties remain unclear and the literature is inconsistent,^6^ especially with respect to the nature of the far field spectral features associated with arrays of apertures in thin metal films.

In this work we present new methods for efficient fabrication of plasmonic nanopore arrays with excellent structure control. We use EBL with a negative resist^25^ to make arrays of nanopores in a membrane such that metallic apertures can be formed before the etch step that defines the pores in the membrane, thereby eliminating the risk of having metal on the walls in the membrane. Also, a new CL method is introduced which removes the risk of breaking the membranes during lift-off. We investigate pores of different diameters, even below 100 nm, with either one or two thin gold films on silicon nitride membranes. The new precise fabrication methods make it possible to elucidate how ordering (long-range *vs.* short-range) of identical pores influences the optical resonance. We show that the short-range ordered arrays behave similarly to the long-range ordered ones but with a few important differences. Finite difference time domain (FDTD) simulations confirm the findings. This study presents new methods for preparing plasmonic nanopore arrays in a well-controlled and highly reproducible manner and results that provide further understanding of their optical properties.

## Results and discussion

**Fig. 1:**
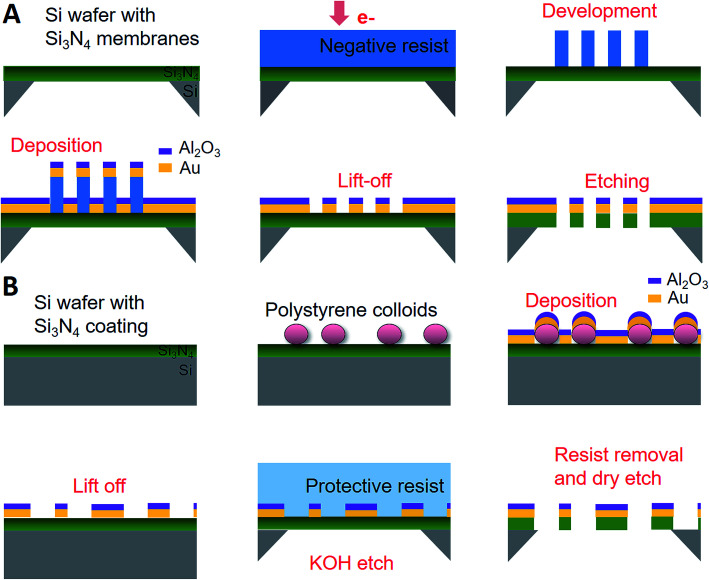
New methods for fabrication of plasmonic nanopore arrays. The most important steps are illustrated. (A) Electron beam lithography with negative resist. (B) Colloidal lithography with protective resist and lift-off before membrane formation. For both methods, an additional gold film can be deposited on the lower side of the membrane before the last etching step.

Fig. 1A shows the main steps for plasmonic nanopore array fabrication by EBL with negative resist. Some examples of challenges when working with negative resists are to ensure their adhesion to the support, creating vertical walls and obtaining rigid structures after development. By fine-tuning parameters such as resist thickness and dose we managed to get ∼300 nm high pillars with highly vertical walls after development. As the lithography is performed on the membranes, which are largely transparent to electrons (“TEM windows”), backscattered electrons from the solid support are avoided. The diameter could be tuned down to ∼70 nm, at which point the pillars started to collapse after development (see ESI†). Smaller pillar (pore) diameters could potentially be obtained by reducing resist thickness and/or changing dose. Notably, entire 4 inch wafers were processed at once, patterning many membranes relatively fast by writing thousands of pillars per second. After the arrays of pillars has been created by EBL, the remaining steps are the same as in our previous work based on CL. A thin gold film is deposited together with a protective alumina (Al_2_O_3_) layer which acts as a mask in the subsequent dry etching.^17^ However, one critical step in the lift-off after deposition which needs to be done under agitation and with the wafer upside down in order to prevent the metal on top of the pillars to land on the membrane top surface. For making pores with double gold films, physical vapor deposition is performed onto the membrane backside before the dry etching. An additional Ar milling step is performed to etch through the bottom gold layer.^17^ The gold film thickness was always 30 nm in this study and hence the structures are semi-transparent.

Fig. 1B shows the new approach for plasmonic nanopore array fabrication by CL, where the key step is to use a protective resist during the KOH etching which defines the membranes. Membranes can break during the mechanical lift-off of colloids,^17^ the exact yield being dependent on membrane dimensions and inherent mechanical stress. We found that especially when the colloids have been reduced in size by O_2_ plasma, after which a higher force is required to remove them,^16^ a majority of membranes broke during lift-off. This led us to try to circumvent the problem by performing the colloid lift-off on the whole Si wafer before forming the membranes^11^ and using a protective resist during KOH etching. Note that the lithography step which defines the openings on the opposite side of the wafer^11,17^ is not included in Fig. 1B. It is critical that the resist which protects the top side of the wafer is covering the surface fully since contact with concentrated KOH will destroy the nanostructure. This implies a thick protective layer, which at the same time should be possible to remove later by solvents or oxygen plasma. We emphasize that the EBL and the CL approach are very similar in the end: the colloids are essentially replaced by a pillar of solidified negative resist, thereby making the shape of the nanopores practically identical regardless of which method is used.

**Fig. 2:**
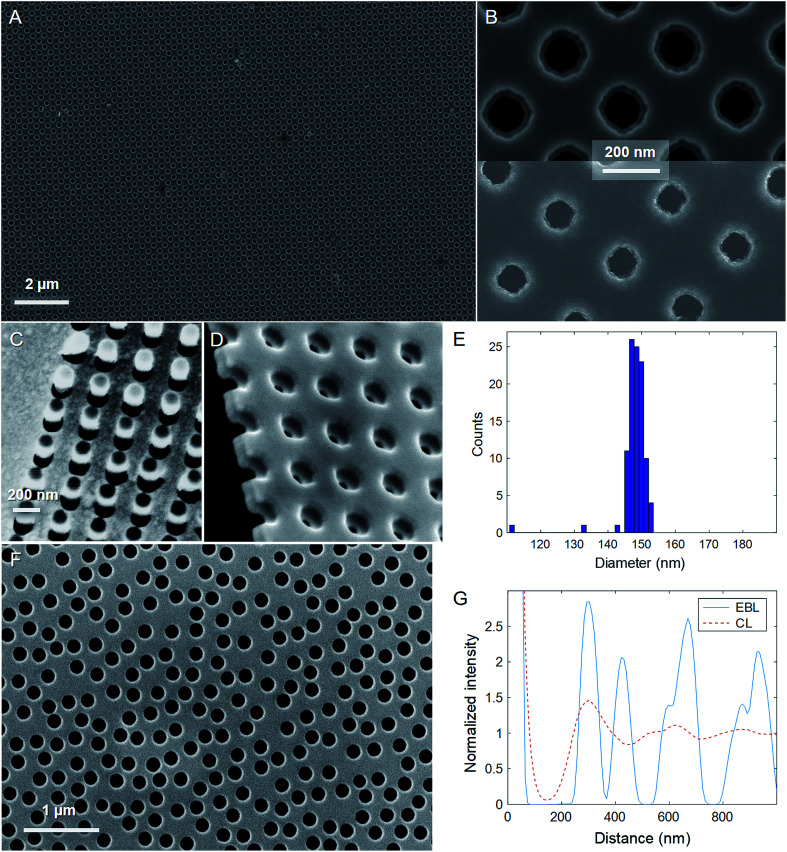
Electron microscopy characterization. (A) Representative image of EBL pores after lift-off. (B) Higher magnification images of EBL pores with diameters 150 nm (double gold film) and 80 nm (single gold film). The aperture in the underlying gold film is visible for the 150 nm pores since it has a slightly smaller diameter than the top aperture. (C) EBL sample after deposition of gold and alumina on the negative resist pillars. (D) A broken membrane with EBL pores (two gold films). (E) Example histogram of aperture diameters for an EBL sample (one gold film). The counts below the main population are due to a few defects. (F) CL pores prepared by the new method. (G) Radial distribution functions for EBL and CL pores.

Electron microscopy characterization is summarized in Fig. 2. For the results presented in this study, all EBL arrays had a square lattice with 300 nm periodicity. Very few defect pore sites (∼1%) were found (Fig. 2A) and the shape of the apertures was as close to circular as expected for polycrystalline gold^26^ (Fig. 2B). Fig. 2C shows the negative resist pillars covered with gold and alumina before lift-off at the edge of the patterned array. The image of a broken membrane in Fig. 2D illustrates that no gold is detected on the silicon nitride walls. For the EBL pores, we also determined the diameter distribution (example in Fig. 2E), which showed that the pores were found within an interval of 10 nm. This is an improvement in homogeneity by approximately a factor of two when comparing to the most narrow diameter distribution that can be achieved with CL.^16^ An example of nanopores prepared by the new CL method is shown in Fig. 2F. As expected, these pores appeared identical to those in previous work^11,17^ and their structure was not analyzed in further detail.

Importantly, we also analyzed the radial distribution functions of the EBL and CL pores (Fig. 2G). The CL pores exhibit a peak at 300 nm, representing the characteristic spacing in the short-range ordered pattern. The EBL pores have the same long range period, although they also show more maxima representing other lattice vectors (*e.g.* diagonally). Overall, it is clear that the EBL lithography with negative resist enabled preparation of pore arrays that were structurally very similar to the CL pores, with the only clear difference that they exhibit long range instead of short range order. Even the number density of the CL pores is very similar to the EBL pores (11 pores per μm^2^ for EBL and 10 pores per μm^2^ for CL).

**Fig. 3:**
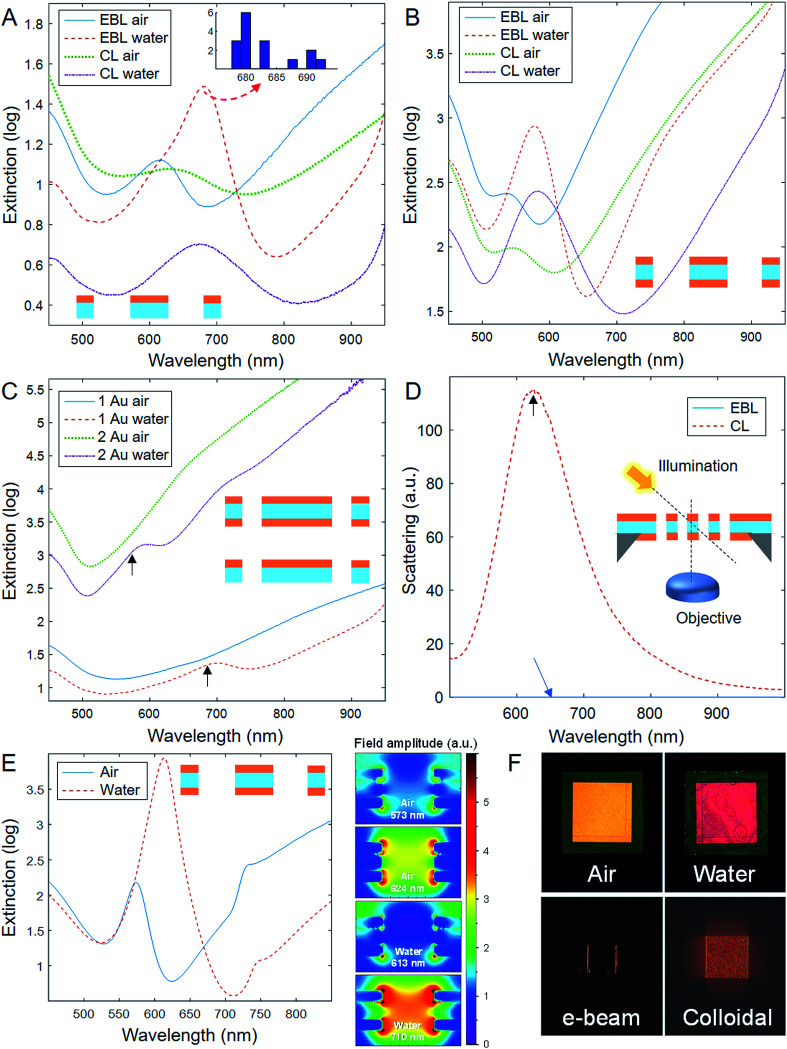
Optical characterization. (A) Extinction spectra for pore arrays with one gold film (diameter 150 nm). The inset shows an example of a histogram of peak positions originating from 16 samples (from different batches). (B) Extinction spectra for pore arrays with two gold films (diameter 150 nm). (C) Extinction spectra of EBL pores with diameter of 80 nm (300 nm periodicity), one or two gold films. The arrows show the peak positions for the same samples but with 150 nm diameter. (D) Scattering spectra from 150 nm pores, two gold films, in air. (E) FDTD simulations of 150 nm pores with two gold films in air and water, including near field plots at the extinction and transmission maxima. (F) Optical camera photos of nanopores with two gold films in transmission illumination (top images, EBL pores in air or water) and dark field illumination (lower images, EBL or CL pores). The membranes are approximately 100 × 100 μm^2^ in all cases.

The optical characterization of EBL and CL pores is presented in Fig. 3. Representative extinction spectra (logarithm of inverse transmission) of 150 nm nanopores in air or water are shown in Fig. 3A (one gold film) and Fig. 3B (two gold films). For all structures, several samples were analyzed (typically ∼10) and found to be reproducible (example of peak position variation in Fig. 3A). As expected, the resonance feature from the nanopore array consists of an extinction maximum (*i.e.* the “peak”) and a transmission maximum (*i.e.* the “dip”) at longer wavelengths.^16–18,21^ The increased extinction in the blue region is due to gold absorption and the increase in the near infrared is due to higher reflection. For the CL pores, the spectra are very similar to previous work and the novelty here is mainly associated with the fabrication process rather than their optical response. We focus instead on the optical properties of the new EBL pores, including how they compare with the CL pores. Fig. 3C shows further spectra of EBL pores where the diameter has been reduced to ∼80 nm (same periodicity of 300 nm). Just like for short-range ordered nanohole arrays,^16^ this leads to a remarkably reduced peak magnitude. In air the resonance features disappear entirely, while in water they appear weak at the same wavelengths as for larger diameters. Fig. 3D compares the scattering response from pores with two gold films prepared by EBL or CL, showing that only the short-range ordered pores give rise to scattering. Fig. 3E shows the simulated extinction spectrum together with the near field distributions at the extinction peak and dip for a 150 nm pore array with two gold films obtained using finite-difference time-domain (FDTD) calculations. The FDTD simulations were performed on the same long-range ordered square lattice as the EBL pores in Fig. 3B. The simulated peak and dip positions are in good agreement with experiments although slightly redshifted (∼20 nm). This may be attributed to small differences in permittivity or the idealized structure used in the simulation compared to the shape of the real pores (Fig. 2B). The calculations also confirmed that the long-range ordered arrays do not scatter light, *i.e.* transmission, reflection and absorption add up to unity (further simulations in ESI†). The small additional resonance feature at ∼730 nm predicted by FDTD calculations is probably due to weak coupling to a lower energy mode. This mode has inversed charge distributions in the individual thin metal films which leads to weaker coupling to the incident light field.^17^ This resonance can actually be observed as a small “bump” in the experimental spectra of EBL pores (Fig. 3). Finally, Fig. 3F shows complimentary photos of the EBL arrays in transmission illumination as well as dark field images of EBL and CL pores.

Considering the optical characterization presented above, we will now discuss how these results contribute to a better understanding of the plasmonic response of nanopore arrays. First, it should be noted that for short-range ordered nanopores (or “nanoholes” for the case of a thicker support), we initially proposed that the extinction peak is originating from excitation of propagating surface plasmons by grating-type coupling (Bloch waves).^27^ This finding has since then been confirmed in various studies, also by several other groups.^19,21,28^ The effect occurs because the apertures in the thin semi-transparent metal films are not random but exhibit short-range order (Fig. 2G) since the colloids cannot adsorb right next to each other due to charge repulsion.^18^ Although previous studies on the effect of the aperture ordering exist, they do have various limitations. Remarkable analogies have been demonstrated for long-range and short-range order in thicker films that have no “ordinary” transmission.^23,24^ However, such opaque films are different because the surface plasmons at the two interfaces are decoupled.^29^ In contrast, thinner films (∼50 nm or less) have hybridized modes with very different dispersion relations.^17^ Even though the effect of short-range *vs.* long-range ordering has been discussed also for nanohole arrays in semi-transparent films,^16,18–20^ previous work has (by necessity) altered the diameter and/or the surface density of apertures. Although interesting, these studies suffer from the fact that many additional effects occur when the void fraction in the metal film is altered.^30^ Our new EBL fabrication with negative resist provides the precision needed to vary only the ordering. As mentioned, from Fig. 2 it is clear that our EBL and CL pores have very similar diameter (a few nm difference) and surface density (<10% difference). Furthermore, the short-range ordered pattern shows a characteristic spacing of 300 nm, matching exactly the periodicity of the EBL pores (Fig. 2G). Indeed, as a result the extinction peak position appears at the same wavelength within the sample to sample variation (less than ±10 nm). Our data shows that this holds regardless of whether the pores are in air or water and even regardless of whether they have one or two gold films. This confirms that the extinction peak is associated with excitation of propagating surface plasmons, with the characteristic pore–pore distance of the CL arrays being analogous to the periodicity of the EBL array.

Nevertheless, when going into details, some differences can be identified between the EBL and the CL pores. The peak is slightly increased in magnitude for the EBL pores, as expected due to more efficient grating coupling to surface plasmons for perfect aperture ordering. However, the most striking effect from disrupting the long-range ordering is arguably the emergence of strong resonant light scattering (Fig. 3D). The periodic arrays only support 0^th^ order reflection and transmission due to the subwavelength distance between pores, while all other diffraction orders become evanescent. Furthermore, scattering at other angles than zero is suppressed due to destructive interference between scattering from different pores of the array.^31^ However, the situation is different for the arrays with short-range order in which the periodicity is perturbed. In turn, the system no longer suppresses non-zero angle scattering *via* destructive interference. This can also be understood as the existence of various spatial frequencies in the structure, giving scattering in more or less in all angles, to be compared with a single hole being like a delta function in space and consisting of all the spatial frequencies. Indeed, scattering is known to occur for single nanoholes in semi-transparent gold films.^32,33^ For nanoparticle arrays prepared by CL, the short-range ordering does contribute to a non-uniform angular scattering pattern.^34^ It was recently demonstrated that scattering can occur also for long-range ordered arrays with comparable diameter and periodicity,^35^ but then the whole array is size-limited (a few μm in total). Similarly, scattering may occur from the edges of the EBL array where the periodicity is interrupted, consistent with the light detected from the array edge in Fig. 3F. We further found that scattering from the CL nanopores with two gold films was even stronger than for pores with a single gold film (roughly four times), which is in agreement with the stronger resonance, *i.e.* more pronounced extinction features, for the double film CL pores (Fig. 3). It may also be related to the role of pore structure in the interplay between metal absorption and scattering as the two possible energy decay paths for surface plasmons.^36^

Next, we will give an explanation why the extinction dip (transmission maximum) is consistently blue shifted for the EBL pores by tens of nm (Fig. 3). Previous work has referred to the transmission maximum as a “localized” resonance,^19,21^ which is correct in the sense that it exhibits behavior characteristic of such modes. For instance, the transmission maximum is very sensitive to pore diameter and shape^16,27^ and has a stronger field enhancement inside the apertures in comparison with the extinction peak^17,20^ as confirmed by the FDTD simulations (Fig. 3E). We believe the picture of two spectral resonances with different properties can be made more generic: In recent theoretical work^29^ we showed how the whole spectral feature of long-range ordered nanohole arrays can be interpreted as Fano interference between the broad transmission *via* single holes in a thin metal film^32,33^ and grating coupling to propagating surface plasmons. Similar behaviour can be expected also for CL arrays, albeit with less efficient grating coupling and correspondingly broader spectral features. Indeed, weakening the resonance strength and broadening the linewidth leads to less steep features for a Fano-shaped “peak and dip” profile and larger spectral distance between peak and dip (calculations in ESI†). Consequently, the red shift of the transmission maximum for the CL pores compared with the EBL pores is in agreement with a Fano model. The weaker peak and dip profiles for smaller diameter (Fig. 3C) are possibly due to the weaker resonance strength and lower direct transmission for reduced pore area, which reduces the contribution of the hole resonance to the continuum background.^29^

Finally, it should be noted that the scattering maximum for the CL pores with two gold films appears very close to the transmission maximum (arrow in Fig. 3D). This differs from data in publications on short-range ordered nanohole arrays in a single gold film, where it is clear that there is resonant scattering at a wavelength approximately in the middle between the extinction peak and dip.^37,38^ This effect can be also understood in the framework of Fano interference. Our recent work has shown that the resonance, if defined in terms of simulated maximum absorption, lies somewhere between the peak and the dip.^29^ When the direct transmission is high (*e.g.* for larger diameter, smaller periodicity, or thinner films), the resonance is positioned close to the extinction maximum, whereas it appears closer to the transmission maximum in case of lower direct transmission (*e.g.* for smaller diameter, larger periodicity, or thicker films). In turn, the lower direct transmission for the nanopores with two gold films, compared to the single gold film samples, results in the resonance being positioned near the transmission maximum. Similar trends were confirmed using FDTD simulations for different hole diameters and different thicknesses of the silicon nitride layer (ESI†). Note that only the short-range ordered arrays scatter light, while only the long-range ordered arrays can be simulated. Hence comparing the simulated absorption maximum with the experimental scattering maximum is the best comparison we can do when determining where the resonance wavelength lies according to the Fano interpretation.

## Conclusions and future outlook

This work has shown a new method for plasmonic nanopore fabrication based on using a negative resist in EBL. The method makes it possible to produce plasmonic nanopore arrays in arbitrary shapes and patterns without any metal ending up on the side walls of the supporting membrane. In addition, we have presented a new method for making (structurally identical) plasmonic nanopore arrays with short-range order by colloidal lithography without any significant risk of breaking the membranes during lift-off. These methods should prove useful for future applications of plasmonic nanopore arrays including sensors with flow-through configuration and/or new smart filters for bioanalytical devices. The possibility of selective chemical modification of different regions of the nanopores is also interesting.^14,39^ In the future, EBL pore patterns of apertures with other shapes than circular, which have attracted attention in theoretical work,^40,41^ may be experimentally investigated.

Furthermore, the fabrication methods have made it possible to present the first comparison of how the optical properties depend on aperture ordering for the case of semi-transparent metal films, maintaining the same diameter and surface density. Only a few differences were identified in the extinction spectra due to long-range aperture ordering: the extinction maximum was increased in magnitude and the transmission maximum was blue shifted by tens of nm. These effects were in agreement with a model that treats the optical resonance of the nanopore arrays as due to Fano interference between direct transmission through single pores in a thin metal film and surface plasmon polaritons in a periodic array. In addition, the strong scattering observed from CL pores can be understood as a diffraction peak of zero order that is broadened due to imperfection of the periodicity. We believe these conclusions fill some of the missing gaps when it comes to understanding the optical properties of aperture arrays in thin metal films, which are now commonly utilized in various applications such as optical manipulation and sensing.

## Author contributions

All authors have contributed to the results and/or their interpretation. Approval of publication of this manuscript has been given by all authors.

## Conflicts of interest

There are no conflicts of interest to declare.

## Supplementary Material

NA-001-C9NA00585D-s001
