# Supplementary material for: Optical properties of plasmonic nanopore arrays prepared by electron beam and colloidal lithography
Source: Nanoscale Adv. 2019 Oct 7;1(11):4282–9. doi: 10.1039/c9na00585d (PMC9418017; doi:10.1039/c9na00585d)
Supplement: NA-001-C9NA00585D-s001 [file NA-001-C9NA00585D-s001.pdf]

Electronic Supplementary Material (ESI) for Nanoscale Advances.
This journal is © The Royal Society of Chemistry 2019

Electronic Supplementary Information

Optical Properties of Plasmonic Nanopore Arrays Prepared by Electron Beam and Colloidal Lithography

*Bita Malekian,[1] Kunli Xiong,[1] Evan Kang,[2] John Andersson,[1] Gustav Emilsson,[1] Marcus Rommel,[3] Takumi Sannomiya,[4] Magnus P. Jonsson[2] and Andreas Dahlin.[1]**

1 Department of Chemistry and Chemical Engineering, Chalmers University of Technology, 41296 Gothenburg, Sweden.

2 Laboratory of Organic Electronics, Linköping University, 60174 Norrköping, Sweden.

3 Department of Microtechnology and Nanoscience, Chalmers University of Technology, 41296 Gothenburg, Sweden.

4 Dept. Materials Science and Engineering, 4259 Nagatsuta Midoriku, Yokohama, 226-8503 Japan.

* Corresponding author: adahlin@chalmers.se

Materials and Methods

Electron beam lithography nanopore fabrication:

The Si [100] wafer was coated by 50 nm Si_3N_4 on both sides using low pressure chemical vapor deposition (LPCVD). A free-standing Si_3N_4 membrane was made using a standard protocol including UV lithography, reactive ion etching and KOH wet etching. The whole 4" wafer was spin coated with Ti Prime (2-methoxy-1-methyl ethyl acetate and 2-metoxipropylacetate) was provided by Sigma-Aldrich as an adhesion layer at 3000 rpm for 20 s. The wafer was then spin coated with negative resist ma-N 2403 (cyclopentanone and anisole) giving ~300 nm resist thickness using 3000 rpm for 1 min, followed by baking in the oven at 110 °C for 10 min. A 100 kV JEOL JBX 9300FS EBL system was employed for writing the patterns in the negative resist. Detailed dose tests were performed to establish the right dose for the given resist thickness. Ma-D 525 (tetramethylammonium hydroxide) was used to dissolve the unexposed (not cross-linked) e-beam resist for 1 min followed by rinsing with water resulting in ~300 nm height pillars. After the development step, 1 nm Cr, 30 nm Au and 15-20 nm Al_2O_3 was deposited on the wafer using e-beam heated physical vapor deposition (Lesker). The metallic coated pillars were then removed during the chemical lift off using mr-Rem 700 (ethanolamine) for at least 4 h under stirring at 55 °C with the wafer positioned with the exposed side facing down in the remover. The resist, the developer and the lift-off solvent were all from Micro Resist Technology GmbH. The Si_3N_4 membrane was then etched using CF_4 and O_2 with a ratio of 4:1 if the structure had no Au film on the other side of the membrane. Otherwise, the backside Au film was first deposited and an additional Ar ion beam milling step was performed to etch through.[1]

Colloidal lithography nanopore fabrication:

Colloidal lithography and shrinking colloids to a desired size were performed on a whole 4’’ Si_3N_4 coated wafer as described in our previous work.[2] The colloids used has a size of 147±7 nm (Microparticles GmbH). After depositing metals, the polystyrene colloids were removed by rubbing the surface while submerged in isopropanol and water. The wafer was then spin coated by photoresist S1813 (Shipley) to protect the nanohole structure during the wet etching of the Si substrate using KOH. The photoresist was then removed by a developer AR 300-76 (Allresist GmbH) after creating the Si_3N_4 membranes. Note that it is important that the resist covers the wafer side with the nanohole array entirely and that it is fully removed after KOH etch. An additional O_2 plasma treatment was done after the developer if resist residues were found. The remaining steps for etching pores through the membrane were performed exactly as for the EBL samples.

Electron microscopy and image analysis:

All the images were obtained using a Zeiss Supra 60 VP. Images were analyzed in MATLAB in order to determine diameters, radial distribution functions and the surface density of holes as follows: (1) Images were filtered using a combination of a gaussian bilateral filter (imbilatfilt) followed by a Wiener filter (wiener2) to reduce noise while preserving edge features. (2) Identification of the centers and radii of pores using circular Hough transform (imfindcircles). (3) Calculation of the radial distribution function (RDF). The RDF for a single pore was calculated using the average intensity of an annulus with a width of 1 pixel, at varying distance from the center of the pore, normalized to the average intensity of the whole image. This was repeated for a maximum of 200 identified pores, to increase calculation speed, and then averaged to obtain the RDF for the analyzed image. The image analysis was performed on inverted images (bright pores on a darker background) such that a normalized intensity greater than 1 indicates correlation.

Spectroscopy:

Microscale extinction spectra were recorded as in previous work[1] using a fiber coupled tungsten lamp and photodiode array spectrometer. A liquid cell with inlet and outlet on both sides of the membrane was used for transmission measurements in water. Images of membranes in transmission were recorded by a Thorlabs camera and dark field images were recorded by a Zeiss camera. Scattering spectra were measured with 10× magnification in air (NA 0.3) using a fiber coupled to a Zeiss microscope output port and a CCD spectrometer (B&WTek). The opening of an SMA fiber from a tungsten light source was used for illumination and placed at an angle of approximately 45° to the membrane plane. The reference spectrum for dark field was measured by collecting scattered light from the rough edge of the borosilicate glass slide on which the sample was placed.

Simulations:

FDTD simulations were performed using Lumerical FDTD Solutions (version 8.15.786). The FDTD simulation size was 300 nm along x and y, and 2000 nm along z. Anti-symmetric, symmetric and perfectly matched layer boundary conditions were applied for x, y and z, respectively, to establish the periodic structure in the xy-plane. The mesh size over the entire material volume was set to be 2 nm for all three axes. The simulation consists of a nitride nanopore structure (n = 2.04) sandwiched by two gold layers of the same structure, in the air (n = 1) or water (n = 1.33) surrounding medium. The permittivity of the gold was given by the built-in material database in the software (Au – Johnson and Christy). A plane wave normal to the film with a wavelength range of 300-1170 nm was used as a source. Transmission (T) and reflection (R) monitors were installed over and under the nanohole array depending on the illumination direction, to calculate the

extinction as $1 - T$ and absorption ($A = 1 - T - R$). Another monitor was additionally installed in the xz-plane ($y = 0$ nm) to obtain the nearfield plots.

Images of collapsed pillars

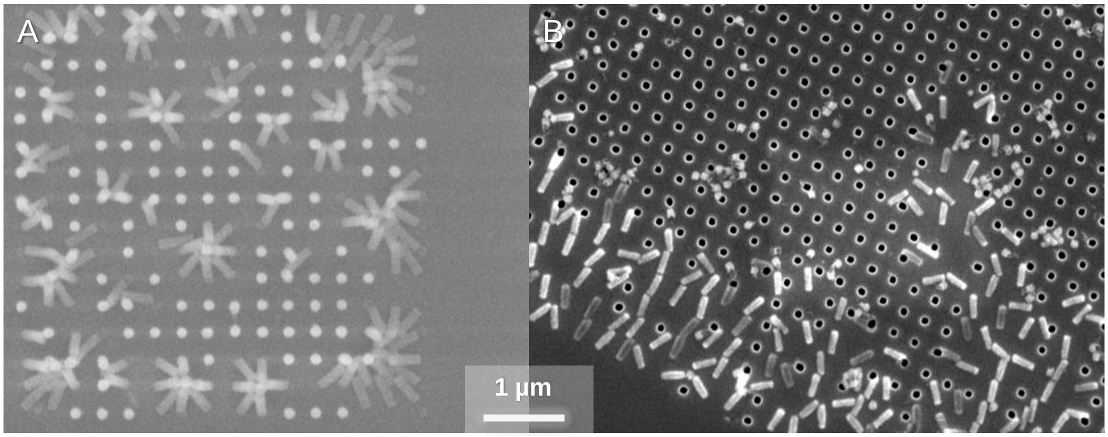

Figure S1. Examples of collapsed negative resist pillars after development (A) and after lift-off (B). The collapse of the pillars occurred when their aspect ratio was too high (here 70 nm diameter and 300 nm height) for a certain electron dose and limits how small the pore diameter can be.

Fano lineshape discussion

Fano interference between the excitation of propagating surface plasmon polaritons and the broad transmission via single holes and a thin metal film contributes to the normalized extinction according to:[3]

$$E_{\mathrm{norm}} = 1 - T_{\mathrm{norm}} = 1 - \frac{1}{1+q^2}\frac{(\varepsilon+q)^2}{1+\varepsilon^2}$$

where q is the Fano asymmetry parameter (known to be negative for nanohole systems), $1/(1+q^2)$ is the normalization constant, $\varepsilon = (E - E_{\mathrm{R}})/(\Gamma_{\mathrm{R}}/2)$ is the reduced energy, E_{R} is the resonance energy and Γ_{R} is the linewidth. The normalized extinction spectra for various linewidths of the resonance and $|q|$ values are depicted in Fig. S2.

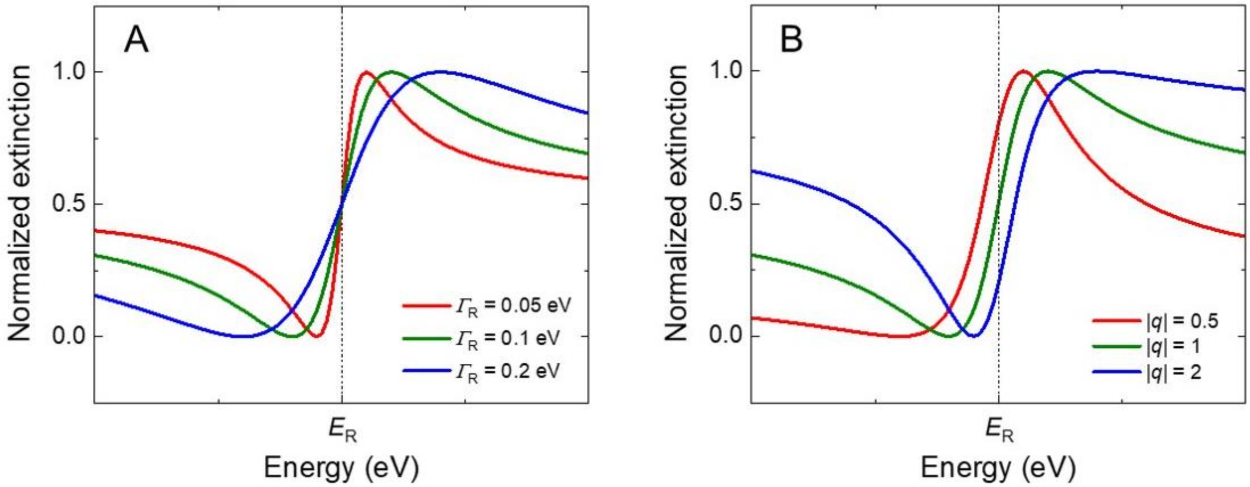

Figure S2. Simulated extinction spectra for (A) various linewidths with $|q| = 1$ and (B) various $|q|$ values with $\Gamma_{\mathrm{R}} = 0.1$ eV.

Additional simulation results

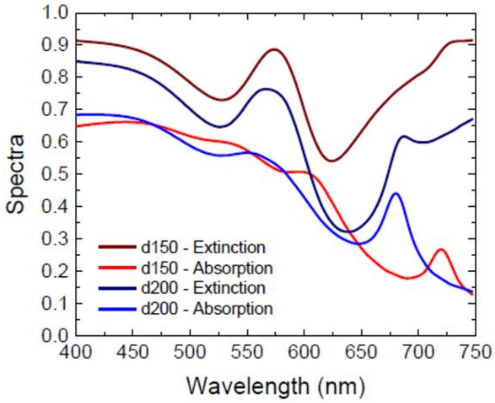

Figure S3. Extinction and absorption spectra for different diameters (*d* in nm) of nanopores with two gold films in air. The extinction peak position is not changed, in agreement with experiments, while the absorption maximum does shift.

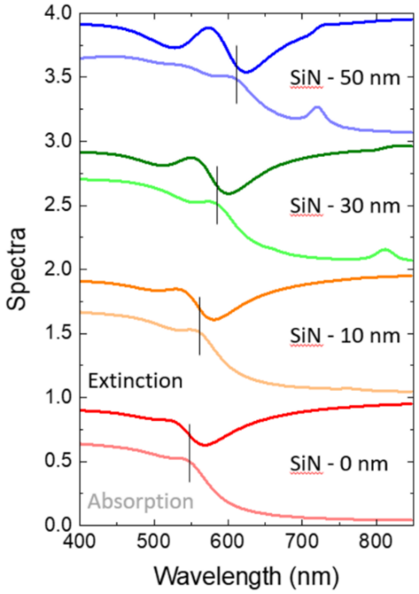

Figure S4. Extinction (dark color) and absorption (light color) spectra for various thickness of the silicon nitride (with two 30 nm gold films). Resonance wavelengths (absorption maximum) are designated by vertical black lines and the spectra are plotted with vertical offsets for better visibility. For 50 nm Si_3N_4 the resonance is very close to the transmission maximum.

References (also cited in main text)

1. A. B. Dahlin, M. Mapar, K. L. Xiong, F. Mazzotta, F. Hook and T. Sannomiya, *Advanced Optical Materials*, 2014, 2, 556-564.
2. K. Xiong, G. Emilsson and A. B. Dahlin, *Analyst*, 2016, 141, 3803-3810.
3. E. S. H. Kang, H. Ekinge and M. P. Jonsson, *Optical Materials Express*, 2019, 9, 1404-1415.
